# Clinical presentation and characteristics of lymphoma in the head and neck region

**DOI:** 10.1186/s13005-018-0186-0

**Published:** 2019-01-03

**Authors:** Katharina Storck, Markus Brandstetter, Ulrich Keller, Andreas Knopf

**Affiliations:** 1 Department of ENT, Head and Neck Surgery, Klinikum Rechts der Isar, Technical University of Munich, Ismaninger Strasse 22, 81675 Munich, Germany; 2 Third Department of Internal Medicine, Haematology and Oncology, Klinikum rechts der Isar, Technische Universität München, Ismaningerstr. 22, 81675 Munich, Germany

**Keywords:** Head and neck, Lymphoma, Neck mass, Symptom, Tonsillitis

## Abstract

**Background:**

The study analyses clinical characteristics of histologically defined head and neck (H&N) lymphoma to raise the awareness of ENT specialists to the leading symptoms.

**Method:**

From 2003 to 2011, all patients with histologically defined H&N lymphoma from our clinic were evaluated.

**Results:**

This study identified 221 patients with H&N lymphoma comprising 193 non-Hodgkin lymphomas (NHL) and 28 Hodgkin lymphomas (HL). Among NHL there were 77 indolent (iNHL), 110 aggressive (aNHL), six highly aggressive NHL and further 28 HL. Patients with highly aggressive NHL and HL were significantly younger (*p* < 0.0001). Corresponding to the leading symptoms, we found nodal and extranodal involvement. NHL demonstrated manifestation in neck lymph nodes, tonsils, major salivary glands, sinonasal-system and hypopharynx/larynx. HL showed exclusive manifestation in lymph nodes of the neck and the tonsils (*p* < 0.0001). The mean time from first symptoms to diagnosis ranged from 1.5 ± 0.7 months in highly aggressive lymphoma to 7.5 ± 11.5 months in iNHL.

**Conclusions:**

The variable clinical presentation of lymphoma is a challenge for the ENT specialist. Fast diagnosis is crucial for rapid treatment, especially in highly aggressive NHL like the Burkitt-lymphoma and HL. A standardized medical history, clinical examination and imaging evaluations paired with patient’s signs, symptoms and demographic knowledge might indicate lymphoma. Biopsies in the H&N region should always be immediately performed in suspicious findings.

## Background

Lymphomas are a heterogeneous group of malignant tumours of the haematopoietic system and are characterized by the aberrant proliferation of mature lymphoid cells or their precursors [1]. Lymphomas can be divided into two major entities: Hodgkin’s lymphoma (HL) and non-Hodgkin’s lymphoma (NHL). Over 20 different subtypes of NHL have been classified according to the specific subtype of lymphoid cells involved.

Several classifications have been developed over the years for lymphomas. The currently used classification is that of the World Health Organization (WHO) and is based on the principles of the Revised European-American Classification of Lymphoid Neoplasm (REAL) from 1994 [2]. The latest update of the classification was published in two reviews in *Blood in 2016* [3–5]. The subtype of the lymphomas is defined based on the cell of origin: B-cell lymphomas, T-cell and natural killer-cell lymphomas (T/NK-NHL) and HL [6, 7]. The two recent WHO classifications from 2008 and 2016 include and encompass (as previously stated in previous classifications) morphology, immunophenotype, genetic and clinical features in order to define “real” diseases [3, 4]. HLs frequently involve lymph nodes of the neck and mediastinum, whereas extranodal sites account for only 5% of HLs for example in the tonsils. In contrast, approximately 30% of NHLs show heterogeneous extranodal manifestations, such as in the major salivary glands, paranasal sinuses, mandible, maxilla and Waldeyer’s ring (largely depending and often characteristic for the specific NHL subtype) [3, 8]. Other than the gastrointestinal tract, the head and neck region is frequently involved as an extranodal site in NHL, affecting 11–33% of patients [9]. The clinical behaviour and manifestations of lymphomas in the head and neck region usually lack specific characteristics that would enable attribution to a specific lymphoma entity without biopsy and histological evidence. In particular, with regard to lymphomas having an aggressive course, immediate histological evidence is crucial for patient management, early treatment initiation and often for the outcome [10, 11]. Available imaging techniques (ultrasound, computed tomography (CT), magnetic resonance imaging (MRI) and positron emission tomography (PET)) fail to distinguish HL from NHL and cannot differentiate their various subtypes, necessitating pathological diagnosis [8]. Sometimes, clinical parameters and the various sites within the head and neck can help to distinguish between the two categories as they each have predilections as mentioned above [3]. Typical symptoms can include an indolent lymphadenopathy (here, we concentrate on the cervical lymph nodes), fatigue, occasionally B-symptoms such as fever > 38 °C, night sweats and weight loss (> 10% within 6 month), susceptibility to infections and changes in the haemogram. Especially with respect to the differential blood count, iNHL presents cytopenia more often than aNHL but it does not lead to the diagnosis as a single parameter. In chronic lymphatic leukemia (CLL), for example, the frequency of lymphocytes in the differential blood count is elevated as a characteristic sign. Further symptoms of lymphoma might include anaemia, leucopenia/leucocytosis and thrombopenia, although specific serum and blood parameters might sometimes also suggest indolent vs aggressive lymphoma, e.g. elevated lactate dehydrogenase (LDH) in cases of highly proliferative disease or increased β2-microglobulin. The most important differential diagnosis for head and neck lymphadenopathy is infection or lymph-node metastasis from regional or distant primaries being affected by solid cancer.

Our retrospective study includes 221 patients who were suffering from NHL and HL and who were consecutively diagnosed in the Department of Otorhinolaryngology, Head and Neck surgery. Histologically confirmed lymphomas were classified according to the clinical system defined below. Thorough analysis of epidemiological data, leading symptomatology, clinical disease presentation and laboratory testing were carried out to identify clinical parameters in order to expedite diagnostic regimes/work-up.

## Patients and methods

All patients presenting with head and neck symptoms that resulted in the histologically established diagnosis of lymphoma from January 2003 to December 2011 (*n* = 221) were included in this retrospective study. The study has been approved by the ethic committee of the Technical University of Munich (Permit Number: 493/17).

A standardized medical history was obtained from all patients: clinical examination, age at diagnosis, gender, location in the head and neck region, imaging evaluations (especially ultrasound), leading symptoms (B-symptoms; fever, night sweats, weight loss), time to diagnosis, known risk factors (HIV, EBV), histological findings and survival outcome (Munich cancer centre). All patients underwent clinical examination and a high-resolution B-mode ultrasound of the neck (S2000, tissue harmonic imaging, 9 MHz linear array, Siemens, Germany). Blood chemistry (including LDH and C-reactive protein (CRP)) and a complete blood count with a leucocyte differential count were undertaken. During diagnostic work-up (staging), all patients also underwent contrast CT-scans of the neck, chest, abdomen and pelvic cavity and bone marrow biopsy. If central nervous system involvement was suspected, staging also included contrast MR imaging of the brain and/or the spine.

**Fig. 1:**
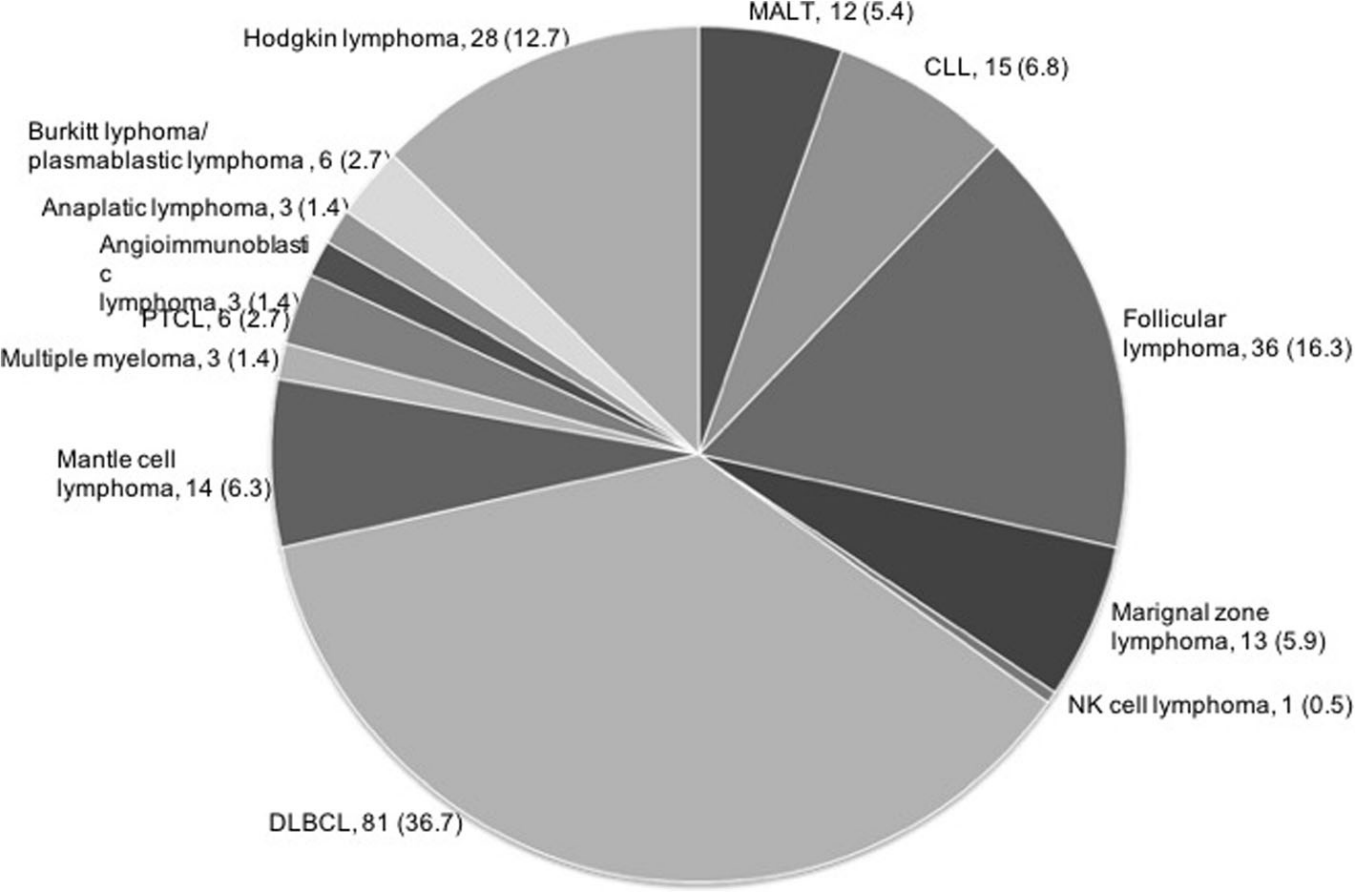
Frequency of histologic subtypes within the iNHL, aNHL and highly aggressive lymphoma and HL

Lymphomas were classified according to the lymphoma classification effective at the time: up until 2008, we used the Revised European American Lymphoma Classification (REAL) [2], and starting from 2008, we employed the WHO classification [3, 4]. For practical purposes and because the observation period spanned two classifications, we refer here to lymphomas in two main categories, namely Hodgkin lymphomas (HL) and non-Hodgkin lymphomas (NHL). NHL were further clinically subgrouped into 1. indolent lymphoma (iNHL) (including follicular lymphomas and margional zone lymphomas), 2. aggressive lymphoma (aNHL) (e.g. DLBCL) and 3. highly aggressive lymphoma (Burkitt lymphoma and lymphoblastic lymphomas) (Fig. 1).

Statistical analyses were performed by using unpaired t-tests and one-way ANOVA testing (SPSS Inc., Chicago, IL). Post-hoc analysis was carried out with Tukey’s test. A *p* value of < 0.05 was considered statistically significant and a *p* value < 0.001 was defined as highly significant.

## Results

### Epidemiology and characteristics of the head and neck cohort

A total of 221 patients were included in this study: 193 with NHL and 28 with HL. With respect to the clinical classification system, we included 77 indolent NHL (iNHL), 110 aggressive lymphomas (aNHL), 6 highly aggressive lymphomas and 28 HL (Fig. 1). The median age for indolent and for aggressive lymphoma was 70 years, for highly aggressive lymphoma 34 years and for HL 33 years. Patients with highly aggressive lymphoma and HL were significantly younger than their counterparts with less aggressive types (*p* < 0.0001; Table 1). The study comprised 114 [52%] males and 107 [48%] females without differences between the groups (Table 1).

**Table 1:** Frequency of histologic types of head and neck lymphoma including also epidemiology, symptomatology, disease manifestations, localization and laboratory findings

	Indolent lymphoma	Aggressive lymphoma	Highly aggressive lymphoma	Hodgkin lymphoma	*p*-value
n	77	110	6	28	
Age					< 0.0001
Mean ± SD	72 ± 39	66 ± 16	41 ± 24	41 ± 21	
Median	70	70	34	33	
Gender, n [%]					0.11
Male	37 [48]	57 [52]	6 [100]	14 [50]	
Female	40 [52]	53 [48]	0	14 [50]	
First Diagnosis, n [%]	62 [81]	90 [82]	5 [83]	27 [96]	0.26
Time to diagnosis, [Months]					0.22
Mean ± SD	7.5 ± 11.5	3.7 ± 8.5	1.5 ± 0.7	3.4 ± 3.5	
Median	3.0	2.0	1.5	1.5	
Leading symptom					0.045
Cervical mass	62 [81]	76 [69]	3 [100]	27 [96]	
Globus pharyngis	3 [4]	6 [5]	0	0	
Odyno−/dysphagia	7 [9]	21 [19]	3 [100]	1 [4]	
Dysphonia	0	1 [1]	0	0	
Dyspnea	2 [3]	3 [3]	0	0	
Incidentally	2 [3]	3 [3]	0	0	
Localization, n [%]					< 0.0001
Major salivary gland	21 [27]	12 [11]	0	0	
Sinonasal system	2 [3]	4 [4]	0	0	
Tonsils	15 [20]	41 [37]	3 [50]	1 [4]	
Hypopharynx/Larynx	1 [1]	6 [6]	0	0	
Lymph node	37 [48]	43 [39]	3 [50]	27 [96]	
Other	1 [1]	4 [4]	0	0	
Laterality, n [%]					0.62
Unilateral	65 [85]	90 [82]	6 [100]	22 [79]	
Bilateral	12 [15]	20 [18]	0	6 [21]	
Systemic disease, n [%]	43 [56]	75 [68]	6 [100]	22 [79]	0.90
B-symptoms, n [%]	5 [7]	20 [18]	0	3 [11]	0.30
Laboratory parameter, Mean ± SD					
Leucocytes	11.8 ± 12.0	7.7 ± 3.6	4.5 ± 3.2	8.0 ± 3.0	0.043
Hemoglobin	13.6 ± 1.9	13.4 ± 2.3	10.4 ± 6.1	13.0 ± 2.9	0.24
CRP	1.53 ± 2.88	1.80 ± 3.00	1.20 ± 2.00	3.30 ± 4.30	0.15
LDH	263 ± 285	278 ± 291	232 ± 229	271 ± 121	0.97

*aNHL* Aggressive non-Hodgkin lymphoma, *haNHL* Highly aggressive non-Hodgkin lymphoma, *HL* Hodgkin lymphoma, *iNHL* Indolent non-Hodgkin lymphoma

The mean time from first symptoms to diagnosis ranged from 1.5 ± 0.7 months in highly aggressive lymphoma to 7.5 ± 11.5 months in indolent lymphoma. This difference was not statistically significant (Table 1).

### Symptoms

Independent from the classification the vast majority of lymphoma patients (*n* = 168) suffered from cervical masses as the leading symptom. Fifty-nine patients complained of odyno−/dysphagia. Globus pharyngis, dysphonia and dyspnea occurred infrequently. Occult lymphoma without clinical symptoms was diagnosed in five patients during sonographic procedure for another disease (Table 1). The distribution of leading symptoms differed significantly between the groups (*p* < 0.05, Table 1). Whereas patients with highly aggressive lymphoma and HL usually presented with a cervical mass and/or odyno/dysphagia, patients with indolent and aggressive lymphoma demonstrated a broad variety of leading symptoms (Table 1). B-symptoms occurred in 28 (13%) patients (NHL, *n* = 25; HL, *n* = 3).

### Disease manifestation

Corresponding to the diverse leading symptoms, we found a nodal and an extranodal involvement of the head and neck organs. NHL demonstrated manifestation in neck lymph nodes (*n* = 83), tonsils (*n* = 60), major salivary glands (*n* = 32), the sinonasal system (*n* = 6) and the hypopharynx/larynx (*n* = 7) whereas HL showed exclusive manifestation in neck lymph nodes (*n* = 27) and the tonsils (*n* = 1). Although highly aggressive NHL and HL exclusively originated in indolent neck lymph nodes and the tonsils, indolent and aggressive lymphomas showed a distinct disease heterotopia (*p* < 0.0001; Table 1). In our study, extranodal head and neck manifestation occurred in 57% NHL and in 4% HL. NHL presented a unilateral localization in 84% of cases, and HLs were unilateral in 79% of cases. We found *n* = 12 cases (20%) of NHL with bilaterally affected tonsils. Systemic involvement was seen in *n* = 43 [56%] patients with iNHL, in *n* = 75 [68%] patients with aNHL, in n = 6 [100%] patients with highly aggressive lymphoma, and in *n* = 22 [79%] patients with HL.

### Laboratory findings

Basic laboratory testing included blood counts, C-reactive protein (CRP) and lactate dehydrogenase (LDH). The level of leucocytes (normal range: 4.0–9.0 [G/l]) differed significantly between the groups (*p* < 0.05; Table 1). However, all levels ranged within the norm. Patients with iNHL exhibited a leucocyte level of 11.8 ± 12.0 (mean ± SD), patients with aNHL 7.7 ± 3.6 and patients with HL 8.0 ± 3.0. In patients with highly aggressive lymphoma, the leucocyte level was significantly decreased at 4.5 ± 3.2. Similar results were seen in haemoglobin levels, which showed a normal level in all the groups, with the lowest level in highly aggressive lymphomas at 10.5 ± 6.1 (mean ± SD). Differences between haemoglobin levels were not statistically significant. CRP (normal range: < 0.5 [mg/dl]) was slightly elevated in all groups, with the highest level in HL at 3.30 ± 4.30 (mean ± SD). LDH levels (normal range: < 244 [U/l]) were elevated in aNHL at 278 ± 291[U/l] (mean ± SD) and in HL at 271 ± 121[U/l] (Table 1).

### Survival outcome

**Fig. 2:**
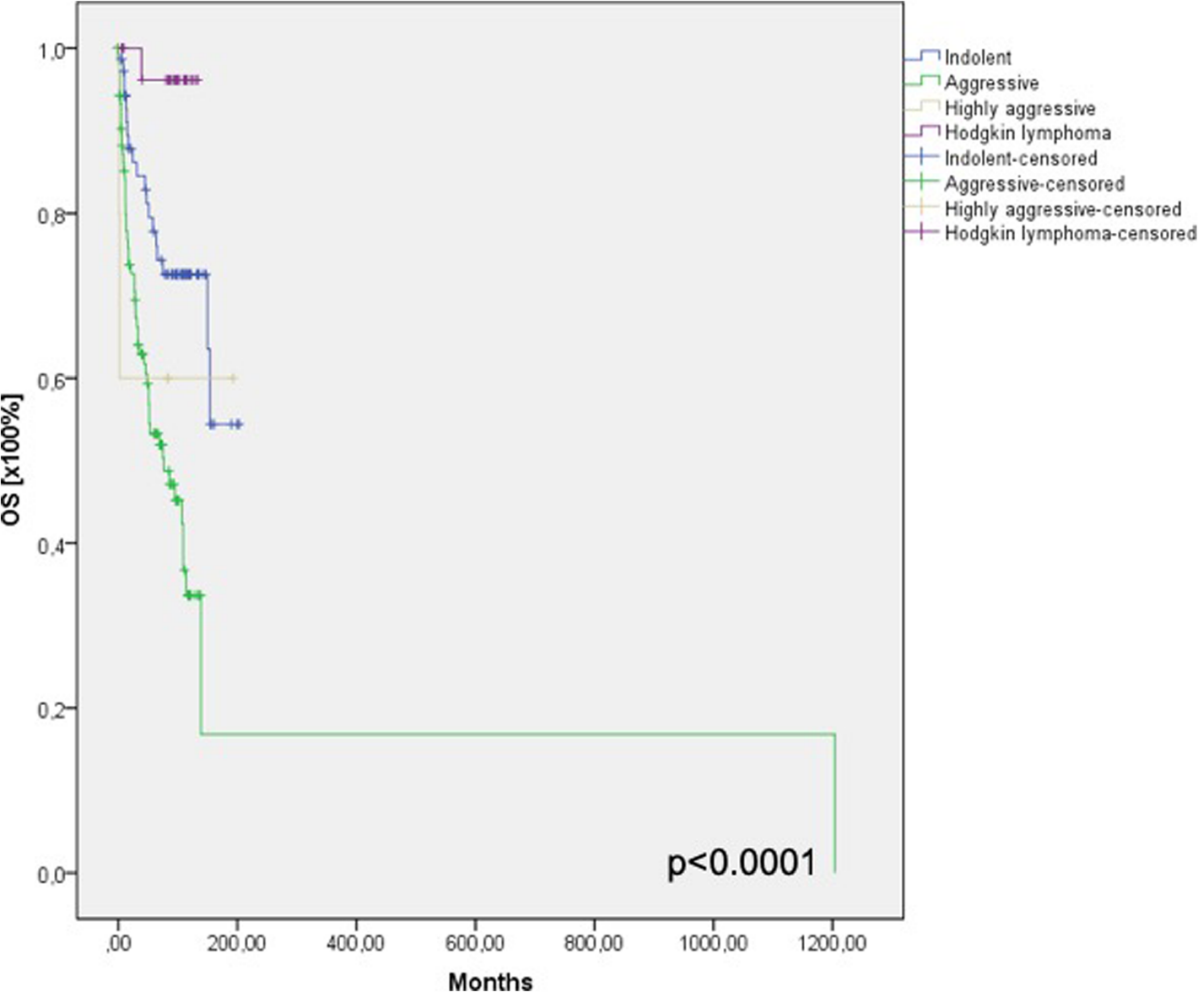
Overall survival data for the previously defined subgroups

Available overall survival data for the previously defined subgroups are shown in Fig. 2.

**Fig. 3:**
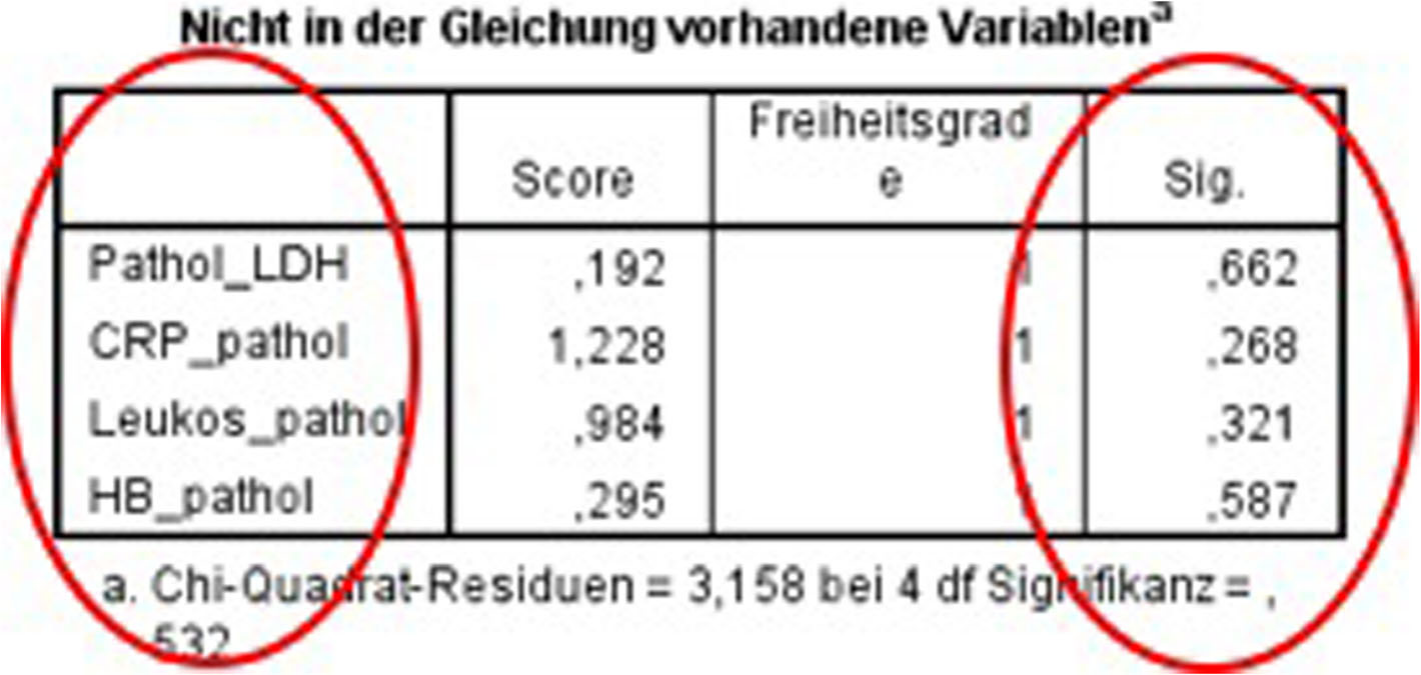
Survival rate depending on the laboratory findings

Using the cox’s regression for forward selection, we also evaluated the survival rate depending on the laboratory findings. We could not find any significant differences in the survival rate depending on (pathological) laboratory findings (Fig. 3).

## Discussion

Lymphoma is the third most common malignancy worldwide representing 3% of all malignant tumours. With 12% of all malignant tumours of the head and neck region, lymphomas are the third most frequent malignancy after squamous cell carcinoma (46%) and thyroid carcinoma (33%) [12, 13] and should thus always be taken into consideration in cases of unknown cervical or oral masses. Misinterpretation of the clinical appearance and of the radiological findings (ultrasound, CT-scan, MRI) can lead to delay in diagnosis, delayed treatment initiation and impairment of the patient’s prognosis. In the current study, we have analysed clinical and epidemiological data of the entire lymphoma cohort diagnosed within an eight-year time frame at our ENT Department in order to bring to the attention of ENT specialists the specific clinical symptoms that allow the early diagnosis of lymphomas. In agreement with recent publications, we saw no differences in the gender distribution. Patients with HL (33 years) and highly aggressive lymphomas (34 years) were significantly younger than patients having other lymphoma subtypes (70 years) [14, 15]. In our study, we found 193 NHL and 28 HL. Concordant with the present literature, diffuse large B-cell NHL (DLBCL) comprised the largest percentage of NHL in the head and neck region with 36.7% of cases [16, 17]. Cervical lymphadenopathy (syn. nodal) is the most common site for both NHL and HL in the head and neck region. Differentiation from other causes of pathological lymph node enlargement caused by infectious diseases (CMV, EBV) or metastatic squamous cell carcinoma is crucial and often difficult and requires histopathological assessment. Certain differences, including the history of alcohol and / or tobacco use, the age of the patient, abnormalities in the clinical ENT examination, constitutional symptoms and systemic lymphadenopathy, may increase the probability of one versus the other. Associated mediastinal adenopathy is more common in HL and abdominal adenopathy in NHL [15]. In our series, we found *n* = 110 (50%) patients presenting with cervical lymphadenopathy. Of these, 90 were unilateral and only 20 presented with a bilateral cervical lymphadenopathy. In HL, 22/27 were unilateral. An important aspect for ENT and maxillofacial specialists is the variety of extranodal sites. Taking all lymphomas together, we found 111 (50%) lymphomas in extranodal sites. In particular, NHL contributed to this high proportion. With *n* = 110 of 193 NHL (57%), NHL presented an extranodal site in a surprisingly large number of cases. In the literature, 25–30% NHL occur in extranodal sites [18]. In HL, we found an extranodal site in just one case (palatine tonsils), whereas all other patients presented with cervical lymph nodes (96%). The literature also describes > 90% manifestations of HL occurring in the lymph nodes and only 1–4% involving extranodal areas [8, 14, 19]. The extranodal sites included in this study were the major salivary glands (*n* = 33), sinonasal system (*n* = 6), palatinal tonsils/nasopharynx (*n* = 60) and hypopharynx/larynx (*n* = 7). The literature also describes extranodal sites such as the palate, buccal mucosa, maxilla and mandible [8, 20]. In our clinic, the patients with lymphomas in bone regions usually attend the Department of Maxillofacial Surgery; thus, patients with extranodal sites are partially preselected. The leading symptoms were correlated with the localization of the tumour mass, the majority of patients presenting with a cervical mass (76%) followed by odyno−/dysphagia, globus pharyngis, dysphonia and dyspnea.

Taking all patients together (*n* = 221), we found that only *n* = 8 (13%) of the patients presented with constitutional symptoms or specific B-symptoms. Only n = 3 patients with HL suffered from B-symptoms. This low percentage agrees with the data in the literature [21, 22]. Concentration on the presence or absence of B-symptoms might thus mislead the physician, as the rate of patients without such symptoms is high. The same also applies to results from blood and serum testing, as the majority of all of our patients had normal haemoglobin and leucocyte counts and only a few had slightly elevated levels of LDH and CRP. According to the WHO classification, two major subtypes of NHL (DLBCL 70–80% and Burkitt 7–20%) are related to HIV [23]. In our cohort, we found two HIV-positive patients. One of them was diagnosed with a nodal DLBCL and the other with nodal plasmablastic lymphoma (PBL), an aggressive and rare DLBCL subtype that is commonly found in patients with HIV [24].

Burkitt lymphoma (BL) is listed in the WHO’s classification of lymphoid tumours as an “aggressive B-cell non-Hodgkin’s lymphoma” characterized by a high degree of proliferation of malignant cells and deregulation of the *MYC* gene [25]. In our study, we found 5 cases of BL and all were male, as reported in the literature [20]; none of them were associated with HIV or EBV [26]. With a median age of 34 years, these patients were significantly younger (*p* < 0.0001) than patients suffering from iNHL or aNHL. Only 1.2% of BL are of extranodal origin in the head and neck [27]. We found three to be extranodal in the tonsils. The median time to diagnosis was 1.5 months. Despite its highly aggressive nature, BL is a curable lymphoma. Patients have a better prognosis when the diagnosis is established rapidly and if they present with a limited stage [28]. BL patients exhibited the lowest leucocyte and haemoglobin levels but the levels still ranged within the norm and the number of patients was too low to make a statistically valid statement. Patients with highly aggressive lymphomas and HL all presented with either a cervical mass as a sign of a nodal lymphoma or odynophagia / dysphagia, with the tonsils as the extranodal site in all cases, reflecting their admission to the ENT Department. These findings were highly significant compared with iNHL and aNHL aggressive lymphomas with a larger variety of localizations and leading symptoms. All six highly aggressive lymphomas showed a unilateral cervical mass but a systemic dissemination.

All Patients received standard therapies through the hematology department or associated hematologists/oncologist.

The focus of this report is to describe the different clinical presentations of lymphomas in the head and neck region and to raise awareness on the wide variety of symptoms. As we included all types of lymphoma that may manifest in the head and neck region, there is a large variety standard therapy approaches which sometimes were also adapted to comorbidity. Thus, the intend of this report was not to focus on this clearly important issue which is covered by numerous publications and results from prospective studies. Furthermore, treatment standards have evolved during the observation time of the patient groups described herein.

Concerning the survival outcome, we could see significant differences between the groups as expected. In the blood and serum testings we did not see any differences concerning the survival rate, which strengthens our assumption that we can not identify lymphomas in the head and neck region only by laboratory findings.

## Conclusion

Lymphomas comprise 12% of all head and neck malignancies. The variable clinical presentation of lymphoma, in addition to the nodal involvement, is sometimes a challenge for the ENT specialist. A rapid diagnosis is crucial for early treatment initiation, especially in cases of BL and HL, which mostly affect younger patients. A standardized medical history, clinical examination and imaging evaluations (especially ultrasound) paired with patient’s signs, symptoms and demographic knowledge (e.g. age, gender, HIV, EBV) may lead to a correct diagnosis and accelerated the decision for a biopsy.

Tumours in the head and neck are easily accessible and a biopsy should immediately be performed following suspicious findings. In particular, for NHL with extranodal involvement in the head and neck occurring at a frequency of 20–30%, biopsy should always be part of the diagnosis in any head and neck lesion, including those in the oral cavity, major salivary glands, oropharynx, nasopharynx, paranasal sinus and larynx. Unilaterality, the absence of EBV or other acute viruses, the absence of an obvious tumour and a systemic involvement (if previously noted at the first presentation) should alert the ENT specialist to lymphomas, even in the absence of B-symptoms or blood disturbances.
